# Supplementary material for: Dual Coating of Chitosan and Albumin Negates the Protein Corona-Induced Reduced Vascular Adhesion of Targeted PLGA Microparticles in Human Blood
Source: Pharmaceutics. 2022 May 9;14(5):1018. doi: 10.3390/pharmaceutics14051018 (PMC9143524; doi:10.3390/pharmaceutics14051018)
Supplement: Supplementary file 1 [file pharmaceutics-14-01018-s001.zip › pharmaceutics-1676371-supplementary.pdf]

Supplemental Materials: Dual Coating of Chitosan and Albumin Negates the Protein Corona-Induced Reduced Vascular Adhesion of Targeted PLGA Microparticles in Human Blood

Supplemental Table S1. Particle size and zeta potential

Particle Type	Size (µm)	Zeta potential (mV)
[U] PLGA	1.6 +/- 0.6	-30.3 +/- 7.3
[U] CSPLGA	1.7 +/- 0.5	+10.7 +/- 2.7
[R] PLGA	1.6 +/- 0.5	-26.2 +/- 3.7
[R] CSPLGA	1.6 +/- 0.5	+7.18 +/- 2.7

The mean particle diameter was obtained from SEM images using Image J. All zeta potential measurements were carried out in deionized water. n ≥ 3. U = unloaded, R = rhodamine-loaded, CS = chitosan.

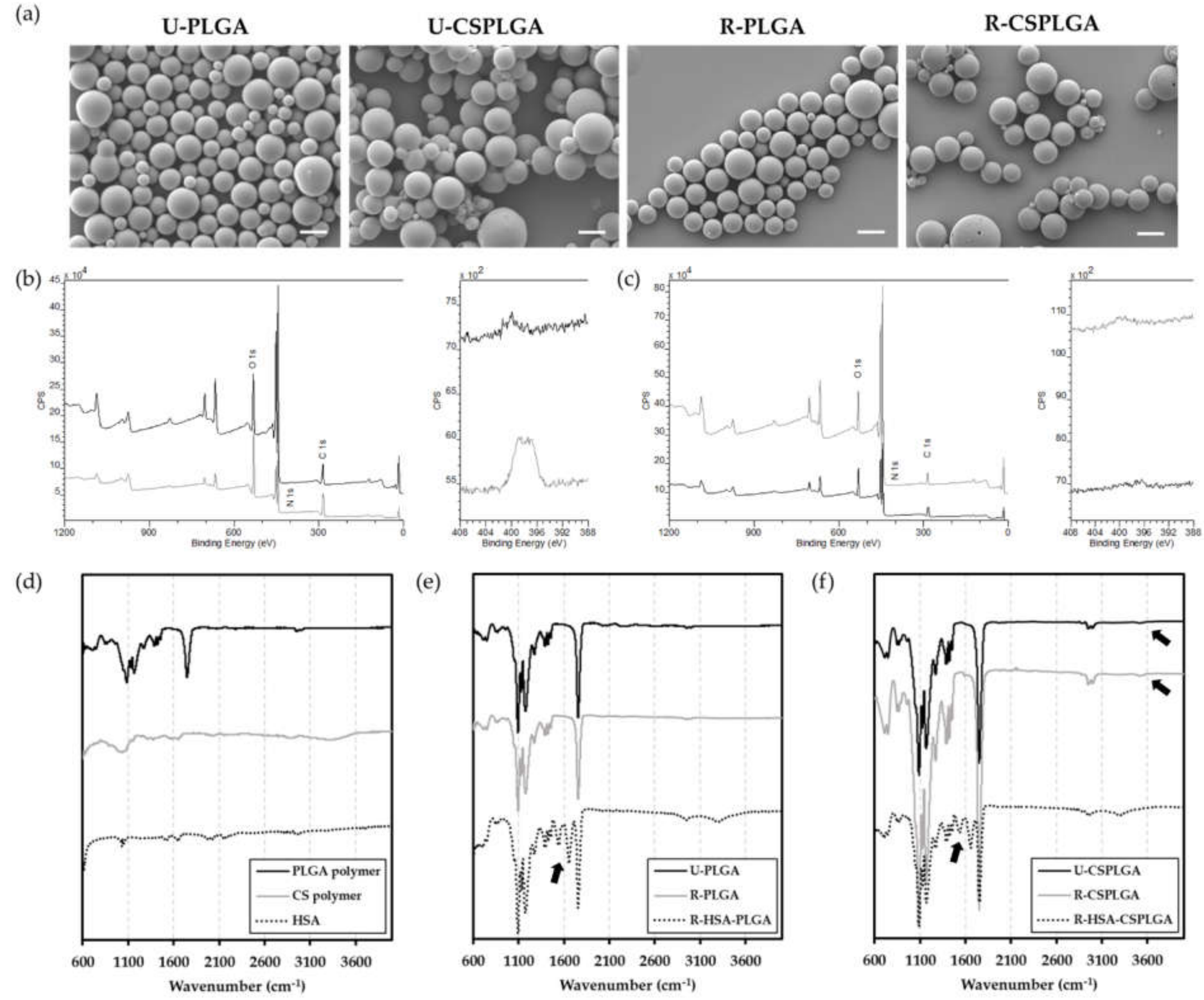

Supplemental Figure S1. Particle surface morphology and chemical characterization. (a) SEM images of unloaded and rhodamine loaded PLGA and CSPLGA. (b) XPS wide spectra of unloaded PLGA (black) and CSPLGA (gray) on the left and N1s spectrum region on the right. (c) XPS wide spectra of rhodamine loaded PLGA (black) and CSPLGA (gray) on the left and N1s spectrum region on the right. (d) FTIR spectra of raw materials. (e) FTIR spectra of PLGA and HSA-PLGA particles. (f) FTIR spectra of CSPLGA and HSA-CSPLGA particles. U = unloaded, R = rhodamine loaded, CS = chitosan, HSA = human serum albumin. Scale bar = 2 μm.

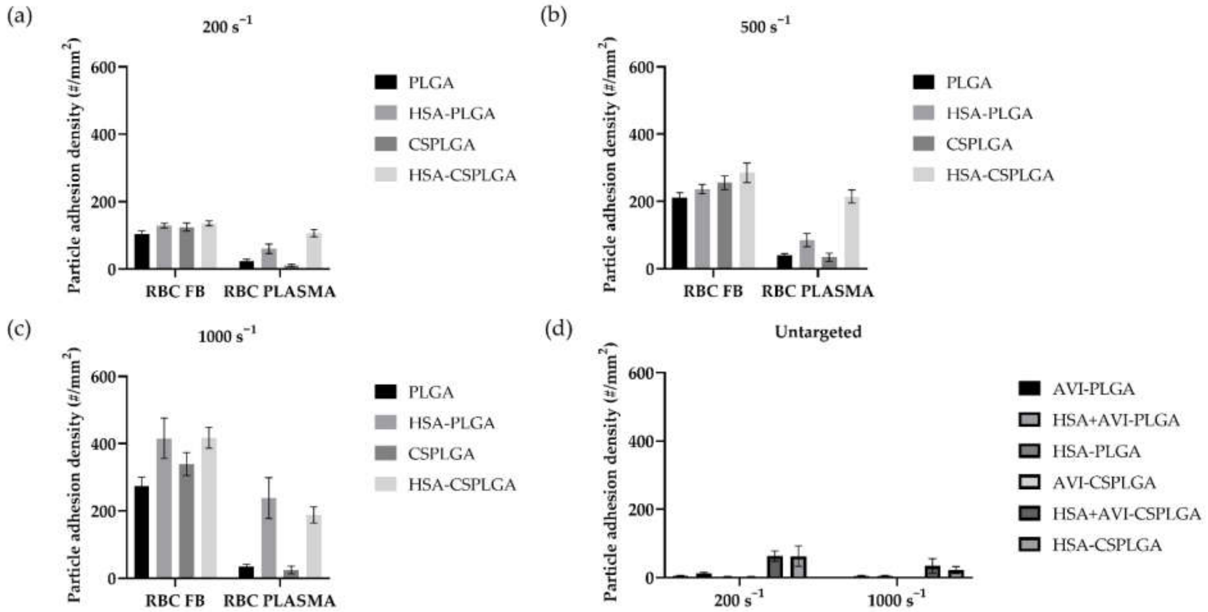

Supplemental Figure S2. Raw particle adhesion density of sLe^A or untargeted PLGA, HSA-PLGA, CSPLGA and HSA-CSPLGA. sLe^A targeted particle binding in RBC in FB and RBC in ACD plasma at 200 s^{-1} (a), 500 s^{-1} (b) and 1000 s^{-1} (c) over endothelium activated for 4 hours. (d) Binding of untargeted particles exposed for RBC in ACD plasma for 5 minutes. n = 10 distinct donors for (a)-(c) and n = 3 for (d).

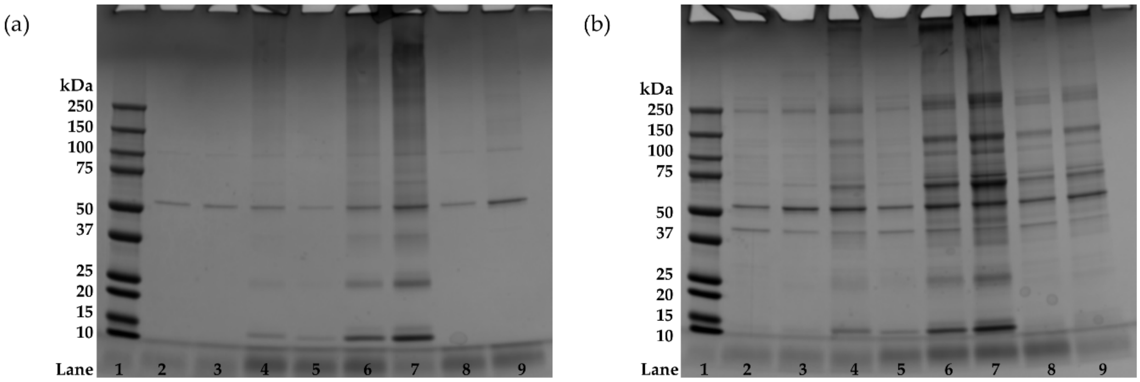

Supplemental Figure S3. Protein adsorption characterization of control and sLe^{A} PLGA or coated PLGA particles. SDS-PAGE on untargeted particles incubated in FB (a) and ACD plasma (b) for 5 min at 37 C. Lane 1: molecular weight ladder, Lane 2: U-PLGA, Lane 3: U-CSPLGA, Lane 4: AVI-PLGA, Lane 5: AVI-CSPLGA, Lane 6: HSA+AVI-PLGA, Lane 7: HSA-+AVI-CSPLGA, Lane 8: HSA-PLGA and Lane 9: HSA-CSPLGA. Lane 1: molecular weight ladder, Lane 2: PLGA, Lane 3: HSA-PLGA, Lane 4: CSPLGA, and Lane 5: HSA-CSPLGA. U = unconjugated, HSA = human serum albumin, AVI = avidin conjugated.

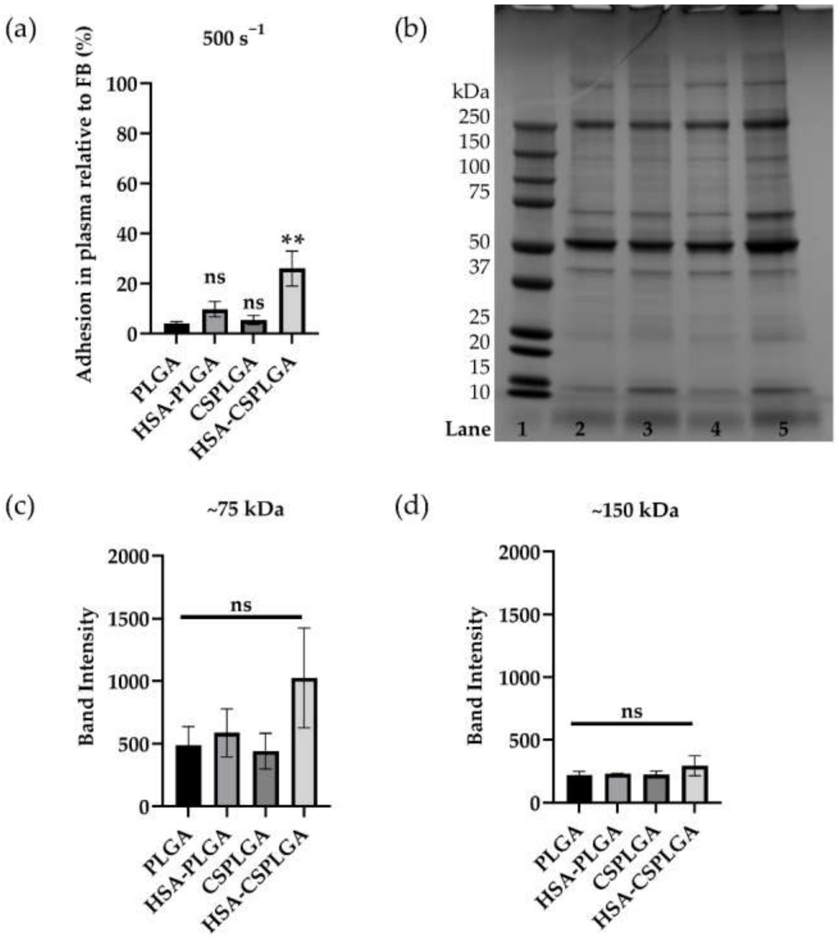

Supplemental Figure S4. Adhesion and protein characterization of sLe^{A} targeted PLGA, HSA-PLGA, CSPLGA, and HSA-CSPLGA in anticoagulant-free plasma at 500 s^{-1}. Particles with 1000 sites/μm^2 of sLe^{A} were incubated in RBC FB and RBC plasma at a 5x10^5 particles/mL concentration and perfused over HUVEC activated for 4 hours. Particles in plasma were added immediately. (a) Particle adhesion efficiency in heparin plasma. (b) SDS-PAGE of particles exposed to anticoagulant-free plasma for 5 min at 37 C. Lane 1: molecular weight ladder, Lane 2: PLGA, Lane 3: HSA-PLGA, Lane 4: CSPLGA, and Lane 5: HSA-CSPLGA. Each lane was analyzed with ImageJ. Plotted are isolated band intensities at ≈75 kDa (c) and ≈150 kDa (d). Statistical analysis was completed using one-way ANOVA with Dunnett's multiple comparison test with PLGA as control: (**) = $p<0.01$ and ns = not significant. n = 7 for A and n = 3 for B-D distinct donors. Error bars represent standard error.
